# Sport-related concussion adopt a more conservative approach to straight path walking and turning during tandem gait

**Published:** 2021-07-16

**Authors:** Nicholas G. Murray, Ryan Moran, Arthur Islas, Phillip Pavilionis, Brian Szekely, Sushma Alphonsa, David Howell, Thomas Buckley, Daniel Cipriani

**Affiliations:** ^1^School of Community Health Sciences, University of Nevada, Reno. 1664 N. Virginia Street, Reno NV 89557, P:(775) 682-8347; ^2^Department of Health Sciences, The University of Alabama, Tuscaloosa. 2103 Capital Hall, Box 870325; ^3^School of Medicine, University of Nevada, Reno. 1664 N. Virginia Street, Reno NV 89557; ^4^Department of Psychology, University of Nevada, Reno. 1664 N. Virginia Street, Reno NV 89557; ^5^Sports Medicine Center, Children’s Hospital Colorado, CO.; Department of Orthopedics, University of Colorado School of Medicine. 13123 East 16^th^ Avenue, Box 060.Aurora, CO 80045; ^6^Department of Kinesiology and Applied Physiology, University of Delaware.; Interdisciplinary programs in Biomechanics and Movement Science, University of Delaware. 349 STAR Tower, 100 Discovery Blvd. Newark, DE 19716; ^7^Doctorate of Physical Therapy Program, West Coast University, Los Angeles. Center for Graduate Studies, 590 North Vermont Ave. Los Angeles, CA 90004

**Keywords:** postural control, TBI, mTBI, sway, center of mass

## Abstract

**Background:**

It is currently unknown what specific neuronal deficits influence postural instability following SRC; however, the modulation of postural control relies heavily on the appropriate integration of sensory information from the visual, vestibular, and somatosensory system. It is possible symptom provocation of vestibular or ocular function is related to unsteady gait patterns during tandem gait.

**Aim:**

The purpose of this study was to evaluate the differences in temporal and center of pressure (CoP) metrics during discrete events of instrumented tandem gait (iTG) among those with sport-related concussion (SRC) compared to healthy controls. Secondarily, this study attempted to evaluate the relationship between iTG CoP metrics and the Vestibular/Ocular Motor Screening (VOMS) Exam.

**Materials and Methods:**

30 collegiate athletes with SRC and 30 healthy controls completed three single task (ST) iTG trials on an instrumented walkway and the VOMS. All individuals with SRC were assessed within 24–48 h post-injury while all controls were measured during pre-participation physicals. CoP metrics in the anteroposterior (AP) and mediolateral (ML) directions and time to completion were evaluated during the first, turn and second pass of iTG between groups. VOMS score was correlated to the CoP metrics across the discrete events.

**Results:**

Athletes with SRC took longer to complete tandem gait (*P*<0.001) along with the first pass, second pass but not the turn when compared to the control group. SRC had slower velocity in the AP direction during both the first (*P*<0.001) and second pass (*P*<0.001) with increased postural sway in the ML direction during the first pass (*P*=0.014). During the turn, athletes with SRC had postural sway in the ML direction (*P*=0.008). Finally, VOMS score was weakly negatively related to CoP velocity in the AP direction during first (*r*=-0.39) and second (r=-0.36) pass while being weakly positively related to postural sway during the turn (*r*=-0.30).

**Conclusions:**

Athletes with SRC adopted a more conservative walking pattern and the presence of vestibular and/or ocular symptoms influence the ability to perform heel-to-toe walking.

**Relevance for patients:**

Individuals with SRC will walk slower during heel-to-toe walking and move more in the ML direction with great movement in the ML direction while en pointe turning. This may increase given the total amount of vestibular or vision symptoms following the SRC.

## 1. Introduction

Sport-related concussions (SRC) are an active public health concern that accounts for 5% to 9% of all sport-related injuries [1]. The signs and symptoms of SRC vary, but postural instability is a cardinal sign of SRC [2]. Postural instability can be assessed numerous ways; however, it is commonly impaired when measured using both static [3] and dynamic [4] instrumented approaches alongside clinical [5-7] tests. Tandem Gait (TG) [7-9] is a clinical dynamic postural task designed to assess the ability to complete a series (single and dual-task) of heel-to-toe walking down and back on a 3 m × 38 mm wide line for time [10]. This time efficient side-line assessment is suggested for use in multimodal assessment of SRC to aid in determining postural instability [11]. Following SRC, the time to complete TG is commonly slower (an average increase of 1.21 s) acutely following an SRC [6-8,12] with an minimally detectable change (MDC) of 0.38 s [13]. When instrumented [8,14], SRC exhibit slower gait velocity, spend more time in double support, and have decreased cadence within 72 h of the injury. These studies indicate that after SRC, individuals complete TG slower, their gait is generally unsteady which is indicative of abnormal postural stability.

It is currently unknown what specific neuronal deficits influence postural instability following SRC, however, the modulation of postural control relies heavily upon the appropriate integration of sensory information from the visual, vestibular, and somatosensory system [15]. The intact system is critical to prevent an unexpected fall during static and dynamic activity. Specifically, sensory system afferent signals converge within the vestibular nuclei and subcortical structures (i.e. substantia nigra pars reticulata) where they are properly coded and integrated [16]. These brain structures receive direct projections from the semicircular canals as well as visual input from the primary visual cortex [16]. While it is unlikely that vestibular organs are damaged from a concussion, research indicates that vestibular [17] and/or ocular function [18] are commonly impaired following SRC. If improper integration occurs from these sensory systems, it may interfere with vestibulospinal tract and reticulospinal projections for lower limb motorneuron activation [19]. This will directly influence the muscular activation and maintenance required for effective postural control which could partially explain the unsteady gait patterns and increased postural sway exhibited during TG post-concussion [20].

Numerous methods exist to measure vestibular or ocular function; however, the Vestibular/Ocular Motor Screening Exam (VOMS) is a commonly used clinical assessment that is low tech, free to use and has high diagnostic validity [17]. The VOMS assess symptom provocation of the vestibular and ocular system via a series of subtests. Those with elevated symptom scores on the VOMS post-concussion, specifically on the Vestibular Ocular Reflex (VOR), may experience delayed concussion recovery [21]. It is unknown why the presence of these symptoms delays recovery, but little is known if symptom provocation of vestibular or ocular function is related to unsteady gait patterns during tandem gait. Research has yet to explore this important relationship from a clinical perspective and it could provide the basis for pursuing more robust, time intensive and methodological techniques.

Thus, the purpose of this research was to evaluate the relationship between instrumented Tandem Gait (iTG) and the VOMS symptom provocation score and near-point convergence (NPC) among those with SRC within 24-48 hours post-injury compared to uninjured control participants. In order to accomplish this purpose, statistically significant group differences in temporal and center of pressure (CoP) metrics during single-task (ST) iTG were examined. Second, these statistically relevant group differences were related to the VOMS total symptom score and NPC. It was hypothesized that deficits in temporal characteristics of iTG will reflect in CoP metrics by a reduction in velocity and increased postural sway. In addition, it is hypothesized that the greater the postural sway, the more provoked symptoms will occur on the VOMS but not increased NPC.

## 2. Methods

### 2.1. Participants

30 NCAA Division I SRC (Female: 20, average age: 20 ± 1 years, average leg length [left and right]: 83 cm, weight: 77 kg) and 30 (Female: 20, average age: 21±1 years, average leg length [left and right]: 83 cm, weight: 70 kg) closely matched controls (CON) participated in the study. Participants were matched on sport (sport position if possible), height, and weight. Concussion diagnosis was determined by the same head team physician within 24 h of the incident using somatic, cognitive, and/or physical self-reported symptoms following an appropriate mechanism (head or body trauma), as well as the Sport Concussion Assessment Tool-5^th^ edition (SCAT-5) [11,22]. Each SRC reported for testing after 12–24 h of rest but was assessed within 24–48 h post-injury. Student-athletes were excluded from the study if they had any self-reported vestibular, visual (excluding corrected myopia or hyperopia through lenses), metabolic, or neurologic pathology (excluding the existing concussion) which included a history of attention deficit hyperactivity disorder, learning disabilities, strabismus or other comparable disorders; chronic injuries (that may have caused time loss from sport participation ≥ 3 months) or any existing lower extremity injury that inhibited the ability to stand/walk. All CON were assessed prior to the beginning of the athletic season. All participants signed informed consent documents and all protocols were approved by the respective site’s institutional review board.

### 2.2. Procedures

Before beginning the testing, anthropometric data such as height, weight, and leg lengths were collected for all student-athletes. Each student-athlete was assessed on the iTG and the Vestibular/Ocular Motor Screening Exam (VOMS) [17]. iTG was administered prior to the VOMS to ensure that dizziness or any other symptoms would not immediately influence the gait protocol.

iTG was recorded using a 3.4 m Tekscan Strideway (30Hz, Tekscan Inc., South Boston, MA), that was calibrated to each individual foot size and pressure distribution before use to adequately measure time and CoP. Three single task (ST) trials and three dual task (DT) trials (serial 7’s) were pseudorandomized and performed [7]. For this particular study, the DT trials were not analyzed as they are a part of larger study. No time limitations were given for the participants but they were encouraged to complete the exam in a timely manner with an attempt to complete it as quickly as possible while still maintaining the heel-to-toe walking pattern. The trials were averaged and further analyzed.

On completion of the iTG, the VOMS was administered. The VOMS [17] is a tool that screens vestibular and ocular motor symptom provocation on various domains. The methods have been published prior [17] and our study used a custom fixation device to standardize the distance the eyes travelled for each item of the exam along with enhancing accuracy for NPC. The custom fixation device consisted of an adjustable, vertical pole affixed to a tripod stand with a leg of the stand that extended to 36″. At the upper end of the vertical pole, a second pole was affixed via a pivot clamp. The length of this pole was 36 inches with 2 white 14 point markers affixed to either end. One end of this part of the prototype contained a secondary pole that had a slide rule device that can be extended out to the end of the nose when aimed at the face to allow for the measurement for NPC. Our preliminary data suggest no differences between the standard VOMS method of administration and using this device at baseline; however, it does reduce the total symptoms provoked following SRC due to standardizing the total distance the eyes must travel during administration [23]. Total symptom provocation was calculated by summing the total number of increased symptoms from baseline (pre-test) for each VOMS item.

### 2.3. Data analysis

The Tekscan Stride way is used to assess gait kinetics across vertical, anteroposterior, and mediolateral directions. The raw force derived from these pressures is transformed to a coordinate system in which the CoP [24] can be calculated [25] and measured during the iTG task. On conclusion of each trial, the raw iTG CoP trajectory in the AP and ML was exported to MATLAB R2019a (Matlab Inc., Natick, MA, USA) where they were manually classified into three discrete events: first pass, turn, and second pass. The first pass was classified as walking the full length of the marked 3-m walkway away from the start point, while the second pass was classified as walking back toward the starting point. The turn was classified as when the student-athletes turned with as few as steps possible at the end of the walkway before the second pass. From these discrete events, time, and CoP trajectory were extrapolated and analyzed further.

The data were assessed for stationary [26], to determine if linear or nonlinear processing techniques needed to be used. This analysis reported that the AP iTG data were linear, while the ML data were nonlinear in nature. Thus for ML signal, empirical mode decomposition (EMD) [27] was performed since this technique is specifically designed to process physiological time series data that have nonlinear signals. Post-EMD mean excursion and velocity were calculated.

Mean excursions in the ML directions were calculated as the sum of the absolute distances between consecutive iTG data points in the entire time-series data divided by the total number of data points. Mean excursion in the AP direction was not calculated due to the participant starting and ending in the same position on the stride way. The following equation demonstrates this calculation Eq. 1:


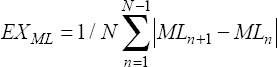


Mean velocity for the AP and ML directions calculated from the absolute difference between iTG excursions values divided by the change time of the time-series data, which was then divided by the total number of data points. The following algorithm expresses this calculation Eq. 2:


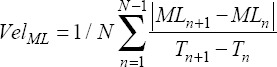


### 2.4. Statistical analysis

All dependent variables were examined for normalcy and to determine if influential skewness exists. None of the time nor CoP metrics were non-parametric, however, the VOMS score was skewed, as expected, due to the control group’s numerous zero values. Thus, a series of multivariate ANOVA (MANOVA) models were constructed in order to compare values between groups on (1) time variables with a 2 (group) × 4 (first pass, second pass, turn time, and total time), (2) mean excursion with a 2 (group) × 3 (first pass, second pass, turn in the ML direction) and (3) mean velocity with a 2 (group) x 5 (first pass, second pass in the ML and AP directions and the turn in the ML direction only). To further determine significant findings, univariate ANOVA were used for the post hoc tests when necessary in the event of a significant MANOVA. A one-way ANOVA was used to determine group differences for NPC and a Kruskal–Wallis test was performed to determine group differences for VOMS change score. Finally, correlation matrices were developed to determine the relationships between significant CoP measures, time measures and VOMS variables. For the VOMS variables, Spearman’s correlation coefficient was used in place of the Pearson’s estimate. The strength of the correlations measures was established as 0.2–0.39=weak, 0.4–0.59=moderate, 0.6-0.79=moderately high, ≥0.8=high. For statistical significance, a *P*-value < 0.05, based on the F-statistic, was established as the critical value.

## 3. Results

### 3.1. Tandem gait time group differences

**Figure 1:**
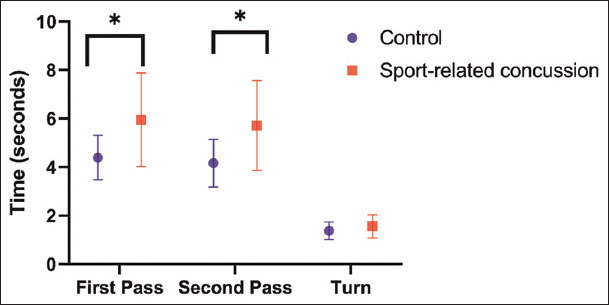
Tandem gait times (mean and standard deviation) for athletes with sport-related concussion and healthy controls for the first pass, second pass, and the turn. The first and second passes were significantly slower for athletes with sport-related concussion but not the turn.

Significant differences between the CON and SRC groups were noted, based on the MANOVA model, for all Time variables (i.e., first pass, second pass, turn, and total time), (*P*<0.001) (Figure 1). SRC were significantly slower on total time (SRC=13.23±3.82s, CON=9.92±2.04s; *P*<0.001, Cohen’s d=1.08) along with the first pass (SRC=5.95±1.94s, CON=4.39±0.91s; *P*<.001, Cohen’s d=1.02) and second pass (SRC=5.71±1.85s, CON=4.16±0.98s; *P*<0.001, Cohen’s d=1.04) (Figure 1). The turn was not significantly different between groups (SRC=1.56±0.48s, CON=1.37±0.36s; *P*=0.08, Cohen’s d=0.48) (Figure 1).

### 3.2. CoP metrics group differences

There was a significant omnibus group effect (*P*=0.008) for CoP mean excursion for the first pass, second pass and the turn in the ML direction. SRC had increased postural sway during the first pass (*P*=0.014) and turn (*P*=0.008), but not during the second pass (*P*=0.490) (Table 1). A significant omnibus group effect (*P*<0.001) for CoP mean velocity for the first pass, second pass in the AP and ML direction along with the turn in the ML direction. SRC had increased postural instability (i.e., increased velocity) for the first pass (*P*<0.001) and second pass (*P*<0.001) in the AP direction, yet no differences were noted between groups on the first pass, second pass, and turn in the ML direction (Table 1).

**Table 1:** Tandem gait center of pressure metrics for the first pass, second pass, and the turn

Variable	Group	Mean (SD)	*P*-value	Cohen’s d
FP Mean Excursion ML (cm)*	Control	1.58 (0.34)	0.014	0.57
	SRC	1.91 (0.62)		
FP Velocity ML (cm/s)	Control	10.35 (2.63)	0.268	0.62
	SRC	9.58 (2.72)		
FP Velocity AP (cm/s)*	Control	69.58 (13.07)	<0.001	1.36
	SRC	54.75 (11.84)		
SP Mean Excursion ML (cm)	Control	1.81 (0.34)	0.491	0.19
	SRC	1.89 (0.48)		
SP Velocity ML (cm/s)	Control	9.48 (2.33)	0.787	0.07
	SRC	9.30 (2.75)		
SP Velocity AP (cm/s)*	Control	72.81 (17.10)	<0.001	1.07
	SRC	56.29 (13.55)		
Turn Mean Excursion ML (cm)*	Control	2.33 (0.68)	0.008	0.73
	SRC	2.85 (0.75)		
Turn Velocity ML	Control	13.45 (4.38)	0.073	0.47
	SRC	15.66 (4.98)		

* Significant group difference; FP: first pass; SP: second pass; ML: mediolateral; AP: anteroposterior; cm: centimeters; SRC: sport-related concussion; SD: standard deviation

### 3.3. VOMS group differences and correlations

There was a significant difference in the VOMS score between the two groups (SRC=11.1±11.2, CON=0.18±.38 symptoms; *P*<0.001, Cohen’s d=1.38); however, the VOMS NPC was not different between the two groups (SRC=4.83±5.88, CON=4.12±4.31cm; *P*<0.001, Cohen’s d=0.13) (Table 2). Using Spearman’s correlation coefficient to account for the non-parametric VOMS score and NPC, significant correlations were noted between the VOMS score and the gait time measures (Table 2); in addition, the VOMS scores were weakly negatively correlated with first pass and second pass velocity in the AP directions while the turn mean excursion was weakly positively related (Table 2).

**Table 2:** Spearman’s rho correlations (R^2^) between VOMS Score and NPC to significant group difference Tandem gait times along with center of pressure variables on the instrumented tandem gait

TG Time	Total Time	First Pass Time	Turn Time	Second Pass Time
VOMS Score	0.41* (0.17)	0.39* (0.15)	0.27* (0.07)	0.39* (0.15)
NPC	0.14 (.02)	0.13 (0.02)	0.26* (0.07)	0.10 (0.01)
**iTG CoP**	**FP Mean Excursion ML**	**FP Velocity AP**	**SP Velocity AP**	**Turn Mean Excursion ML**

VOMS Score	−0.16 (.03)	−0.39* (0.15)	−0.36* (0.13)	0.30* (0.09)
NPC	−0.08 (.01)	−0.15 (0.03)	−0.12 (0.01)	0.13 (0.02)

* Significant correlation at *P*<0.05; FP: first pass; SP: second pass; AP: anteroposterior; ML: mediolateral; VOMS: vestibular ocular motor screening exam; NPC: near point convergence

## 4. Discussion

The purpose of this research was to evaluate the relationship between instrumented tandem gait and the VOMS symptom provocation score and NPC among those with SRC within 24–48 h post-injury compared to uninjured control participants. The findings of this study are that individuals with SRC adopt a more conservative straight path walking strategy during TG. This speculation is supported by a longer completion time, slower AP velocity and greater postural instability in the ML direction during the first pass when compared to healthy controls. The increased sway is indicative of worse dynamic balance control which directly influences the ability to ambulate in a forward direction thus forcing the individual with a concussion to carefully control forward progression to limit a fall. This is similar to prior instrumented [6,14] and non-instrumented TG assessments post-concussion [7,12]. In addition, as anticipated, VOMS scores differed between the SRC group and matched controls, which supports previous literature that reported worse VOMS symptom provocation within 7 days post-concussion while further validating the consistency of the VOMS scores [17,28]. More importantly, as VOMS score increased the time to complete the iTG increased (ranging from weak to moderate positive relationships). Similarly, as VOMS score increased reduced postural stability occurred during straight path walking while increased postural sway was evidenced during the turns. These data suggest that the VOMS score are related, although at times weakly, to time to complete TG and postural instability.

As expected and similar to prior research [7,12], the participants with SRC took significantly more time to complete single-task TG on the entire trial, the first and second pass but not for the turn. These findings are not unexpected given that individuals with SRC typically complete TG slower, but no prior research has analyzed these discrete events. VOMS score was weakly to moderately, positively related to all TG times except for ST Turn (Table 2). These data suggest that the more VOMS symptoms are provoked, the worse SRC typically performs on the timed sections of TG. This could be due to the nature of concussion affecting multiple vestibular domains, including the vestibular-ocular reflex and the vestibulospinal tract [19]. If interrupted, SRC will adopt a more conservative approach to heel-to-toe walking.

In addition, SRC had slower CoP velocity in the AP direction during the first and second pass in the AP direction with increased postural sway during the first pass in the ML direction. These results are partially supported by prior gait and SRC literature [8] where typically forward progression is slower post-concussion as evidenced by a reduced cadence and gait velocity. The trend of increased provoked symptoms on the VOMS and reduced AP velocity during both passes following SRC is not surprising as velocity decreases congruently with increases in TG completion time. It is also speculated that decreased velocity may be concurrent with common impairments of postural stability and unsteadiness 24–48 h post-concussion, especially accompanied by increased symptomatology [29]. The correlation data support this claim as the symptoms on the VOMS were related to reductions in CoP AP velocity and increased ML sway. The VOMS symptoms span somatic and vestibular-ocular domains (i.e., headache and dizziness) which if present can lead to the adoption of a hip strategy to maintain upright position and ambulation [30]. The use of a hip strategy reduces reliance on the ankle flexors, which will diminish forward ambulation and increase ML sway to prevent an unexpected fall. This is further exaggerated in heel-to-toe walking which reduces the overall base of support and increases ML sway yet this is speculative.

During the turn, SRC had significantly greater postural sway in the ML direction while no differences were observed for the velocity metrics. This is supported by prior research [31], as most SRC adopted a larger and slower turn; however, this has not been replicated on a pivot turn. It is likely that during a 180° pivot turn; most healthy controls can execute it without much difficulty en pointe. However, due to the concussions symptoms and possible dynamic postural impairment, SRC have increased sway in the ML direction. This is supported by the weak positive relationship between the turns and the VOMS score (Table 2). It is likely that during the sudden 180º pivot turn, those with higher symptom provocation required more space to complete the turn due to the ocular or vestibular interactions induced from the sudden turn. The presence of symptoms can interfere with proper integration of sensory information which in turn may interfere with efferent vestibulospinal tract and reticulospinal projections for lower limb motorneuron activation [19] which could influence postural stability during locomotion. Further research is needed specifically examining how eye movements or integration of eye and head movements influence the postural stability during before and during the turn.

This research is not without limitations. Not all participants in this study post-SRC demonstrated a clinically meaningful VOMS score (≥2 provoked symptoms). These individuals, while few in number (*n*=5), could have more stable postural control given the lack of provoked symptoms. While it is important to note that the VOMS is reliable as a self-report measure, it is not a true stand-alone vestibular test given its lack of objective data. Future research should compare incorporate eye tracking and/or objective measures of vestibular function such as a modified head-shaking test. Additional limitations for this study are the small sample size and the potential selection bias given the NCAA Division I athletes and inclusion criteria. These aspects will limit generalizability of the findings of this study.

## 5. Conclusion

The results of this study indicate that during iTG, participants with SRC perform all discrete events of TG slower and have slower velocity during the straight path heel-to-toe walking. Additionally, participants with SRC have greater ML postural sway during the turns, which is typical of most neurologic populations during turning. More importantly, the VOMS symptom provocation is weakly to moderately related to the majority of TG times and some iTG CoP metrics. Future research is needed to examine which subtest items of the VOMS relate to time to complete TG and iTG CoP metrics.
